# A Novel Fusion Framework Based on Adaptive PCNN in NSCT Domain for Whole-Body PET and CT Images

**DOI:** 10.1155/2017/8407019

**Published:** 2017-04-03

**Authors:** Zhiying Song, Huiyan Jiang, Siqi Li

**Affiliations:** Software College, Northeastern University, Shenyang 110819, China

## Abstract

The PET and CT fusion images, combining the anatomical and functional information, have important clinical meaning. This paper proposes a novel fusion framework based on adaptive pulse-coupled neural networks (PCNNs) in nonsubsampled contourlet transform (NSCT) domain for fusing whole-body PET and CT images. Firstly, the gradient average of each pixel is chosen as the linking strength of PCNN model to implement self-adaptability. Secondly, to improve the fusion performance, the novel sum-modified Laplacian (NSML) and energy of edge (EOE) are extracted as the external inputs of the PCNN models for low- and high-pass subbands, respectively. Lastly, the rule of max region energy is adopted as the fusion rule and different energy templates are employed in the low- and high-pass subbands. The experimental results on whole-body PET and CT data (239 slices contained by each modality) show that the proposed framework outperforms the other six methods in terms of the seven commonly used fusion performance metrics.

## 1. Introduction

Medical images are very significant to clinical diagnosis and treatment. However, only one modality of image could not provide sufficient clinical information. For example, the Positron Emission Tomography (PET) images only reflect functional information and the Computed Tomography (CT) images only reflect the anatomical information [1]. Therefore, it is necessary to use different modalities of images to provide complementary information to physicians for better diagnosis. Medical image fusion is the process of collaboratively combining the complementary information from multimodal source medical images into one single fused image for further process. The fused image is suitable for visual perception, analysis, and diagnosis which is of great clinical meaning [2, 3].

According to different image domains, image fusion methods are generally classified into two categories: spatial domain-based and transform domain-based methods [4]. The spatial domain-based methods perform on the pixels of the original image directly. They are simple but with poor performance as they would lead to reduction of the contrast and distortion of the spectral characteristics [2]. Currently, the image fusion methods are mainly based on the transform domain, such as discrete wavelet transform (DWT), nonsubsampled shearlet transform (NSST), contourlet transform, and nonsubsampled contourlet transform (NSCT) [1, 4]. By transforming, source images could be decomposed into high- and low-pass subbands. After that, different fusion rules could be designed for different subbands. Finally, the inverse transform would be adopted to obtain the final fusion result.

It is known that the fusion result could be improved by making better use of image information. NSCT is a multiscale, multidirectional, and fully shift-invariant transform which could well suppress pseudo-Gibbs phenomena [5, 6]. Besides, more information could be obtained by NSCT leading to better fusion performance. Thus, NSCT is suitable for image fusion [7–10]. For example, [5] proposed a fusion method based on NSCT which outperformed the DWT-based methods. However, the method did not make enough use of the feature information (e.g., edges, textures) contained by source images as only the gray value of pixel was considered in the low-pass subband. Besides, the fusion rule of low-pass subband, averaging method, could lead to loss of contrast and hence possible cancellation of few patterns in source images. Reference [8] aimed to improve the fusion performance by using maximum entropy of squares of the coefficients in a local window for the low-pass subband and maximum weighted sum-modified Laplacian for the high-pass subbands. Quantitative evaluation results demonstrated that the method was superior to the compared methods. In [10], the average weighting and maximum regional energy rules were adopted as the fusion rules for low- and high-pass subbands, respectively. Results showed the effectiveness of the method on the fusion of images of brains.

However, there is still room to improve for the fusion performance of NSCT-based methods. PCNN, a visual cortex-inspired neural network, involves the global features of the source images that could extract and contain the detailed information effectively [11, 12]. Therefore, many researchers are devoted to combine PCNN with NSCT for a better fusion performance [13–16]. For instance, [13] adopted PCNN in the high-pass subbands of NSCT domain and achieved better fusion results than methods based on Laplacian, DWT, and NSCT. Nevertheless, this PCNN model had lack of adaptability as there were many parameters needed to be adjusted manually. To improve the adaptability, [14] developed a fusion method based on adaptive dual-channel unit-linking PCNN in NSCT domain to decrease the number of parameters and achieved satisfactory fusion performance. Human eyes are sensitive to edge, direction, and texture information rather than single pixels; however, methods in [13, 14] only considered the normalized gray level of image pixels as the external input of PCNN, which could affect the fusion effectiveness to some extent. Reference [15] employed an adaptive PCNN-NSCT based fusion method where the orientation information was utilized as the linking strength in PCNN model. To make better use of edge information, [15] adopted the modified spatial frequency (SF) as the external input of PCNN, which achieved a preferable fusion performance. However, in [15], the PCNN was only adopted in the high-pass subbands where the feature information contained by low-pass subbands was not considered enough.

In conclusion, it is observed that although the PCNN-NSCT based methods could achieve good results, there are still some critical factors which could affect the fusion performance. One is that many parameters need to be set manually in PCNN, that is, lack of adaptability. Another is how to design the external input of PCNN and the fusion rules for more ideal fusion results. To address these issues, in this paper, a modified fusion framework based on PCNN-NSCT is proposed for fusing whole-body PET and CT images. There are mainly three contributions. Firstly, for the sake of implementing the adaptability of PCNN, the average gradient of each pixel is utilized as the linking strength. Secondly, to take better advantages of feature information contained by input images, we use PCNN model in both the low- and high-pass subbands, where the novel sum-modified Laplacian (NSML) and the energy of edge (EOE) are selected as the external input of the PCNN model in low- and high-pass subbands, respectively. This is because NSML [14] could well reflect the edge details of low-pass subband and the EOE [16] could well retain the details of source input as it could denote the edge features in horizontal, vertical, and diagonal directions. Lastly, the region energy is chosen as the fusion rule and two different energy templates are used for the low- and high-pass subbands, respectively. Experiments on whole-body PET and CT images showed the effectiveness of our framework.

The remaining sections of this paper are organized as follows.  Section 2 reviews the theory of NSCT and PCNN.  Section 3 introduces the proposed framework in detail.  Section 4 presents the experimental results and discussion. Finally, the conclusions are given in Section 5.

## 2. Background Knowledge

### 2.1. Nonsubsampled Contourlet Transform (NSCT)

**Figure 1:**
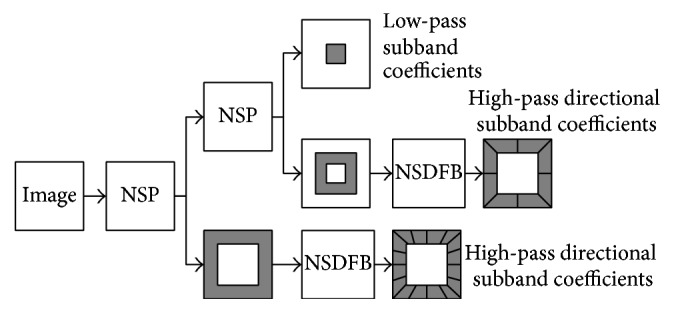
Two-stage decomposition framework of NSCT.

NSCT is a multiscale, multidirectional, and translation invariant transform [17, 18]. Different from contourlet transform [19], NSCT does not employ down-samplers/up-samplers so that it could ensure the translation invariance and could effectively represent the edge and contour information. As a result, the pseudo-Gibbs phenomena could be well overcome which could improve the performance of image fusion. The structure of a NSCT with two-stage decomposition is shown in Figure 1.

As shown in Figure 1, NSCT is composed by nonsubsampled pyramid (NSP) decomposition and nonsubsampled directional filter banks (NSDFB). These two parts could ensure the multiscale property and the multidirectional property, respectively. Firstly, the NSP is performed on the source image to achieve multiscale decomposition. Through NSP decomposition, one low-pass subband and one high-pass subband could be obtained at each NSCT decomposition stage. Then, the NSDFB is employed on the high-pass subbands at each stage to produce high-pass directional coefficients. Through this, the more detailed directional information which is important for fusion could be extracted, with, finally, iterating the former steps on the low-pass subband until the defined decomposition levels are reached. As a result, one low-pass subband coefficient and several high-pass subband coefficients with the same size as the source image could be obtained.

### 2.2. Pulse Couple Neuron Network (PCNN)

**Figure 2:**
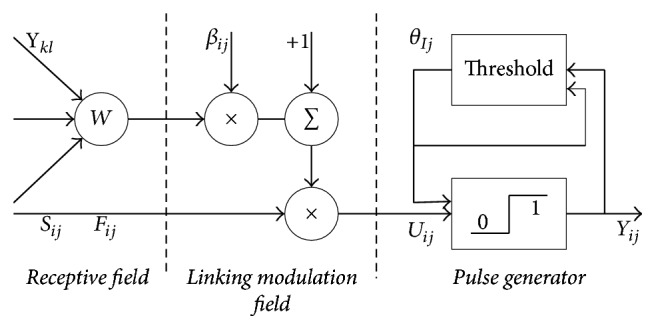
The model of PCNN.

PCNN is a single layered, two-dimensional array of laterally connected network of integrate-and-fire neurons [20]. Each particular neuron corresponds to one particular pixel, which would be also affected by the surrounding neurons. As the traditional PCNN model is complicated, a simplified PCNN model [21] is used in this paper. As shown in Figure 2, a PCNN neuron contains three parts: receptive field, linking modulation field, and pulse generator. Mathematically, the PCNN model could be descripted as

(1) Fijn=SijLijn=exp⁡−αLLijn−1+VL∑klWijklYkln−1Uijn=Fijn1+βLijnθijn=exp⁡−αθθijn−1+VθYijnYijn=1,Uijn≥θij0,Uijn<θij.

Firstly, the input of the neuron consists of two parts: the external input (external stimulus) *S*_*ij*_ and the pulse output of its neighboring neurons *Y*_*kl*_. Traditionally, *S*_*ij*_ is the normalized gray value of the corresponding pixel (*i*, *j*). Subsequently, the nonlinear modulation is performed in the linking modulation filed and then the internal activity *U*_*ij*_ could be obtained. Finally, *U*_*ij*_ is compared with the dynamic threshold *θ*_*ij*_. If *U*_*ij*_ is larger than *θ*_*ij*_, then the neuron would be ignited. In addition, *n* is the total iteration times. (*α*_*L*_, *α*_*θ*_)  and (*V*_*L*_, *V*_*θ*_)  are the time constants and normalizing constants for the linked input and dynamic threshold, respectively. The linking strength *β* reflects the weight of linking field that plays a key role in fusion. Traditionally, *β* is chosen according to experiences which is lack of self-adaptability.

## 3. Our Proposed Fusion Framework

The linking strength *β* plays a key role in PCNN which determines the lifting range and exciting character. It is generally determined manually according to experience which is lack of self-adaptability. In addition, the external input of the PCNN is usually the pixel coefficient in spatial domain or in the transform domain which does not make full use of the edge information of the source image. To address these shortcomings, this paper proposes a novel PCNN-NSCT based fusion framework where the regional average gradient is as the linking strength to achieve self-adaptability. Moreover, the novel sum-modified Laplacian (NSML) and energy of edge (EOE) are calculated as the external inputs for the low- and high-pass PCNNs, respectively. Besides, it is known that the fusion rules determine the fusion effectiveness. Generally, the maximum or averaging rules are used as the fusion rules which will lead to loss of contrast or information. Thus, the rule of max region energy is employed as the fusion rule in this paper. To further improve the fusion effectiveness, different energy templates are used for low- and high-pass subbands.

Since devices that capture CT and PET images are different from each other, it is necessary to correct spatial displacements such as offset, scale, and geometric distortion in advance. In image fusion, the alignment or registration process is important. In this paper, we adopt the registration framework that we proposed in [22] including preprocessing, feature extraction, and registration, which would not be introduced in this paper.

**Figure 3:**
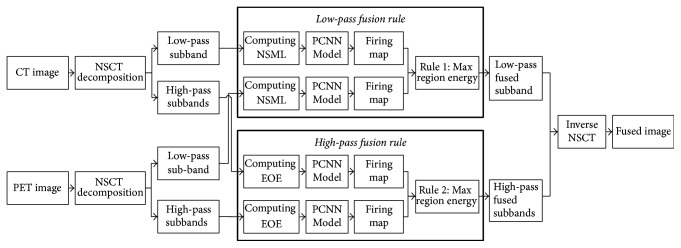
The process flow of the proposed image fusion framework.

Figure 3 illustrates the process flow of our proposed fusion framework. The detailed fusion process consists of the following steps:

1. The NSCT decomposition is performed on both the source PET and CT images. Then one low-pass subband image and a series of high-pass subband images could be obtained.
2. The NSML feature and the EOE feature are computed for the low- and high-pass subbands, respectively. These features would be used as the external inputs of the subsequent PCNN models. After that, both the input coefficients of the PCNN models of the low- and high-pass subbands are normalized to [0, 1].
3. Through PCNN, the firing maps could be produced for these subbands.
4. The firing maps of the low- and high-pass subbands could be fused according to the rule of max region energy. Note that different energy templates are used in low- and high-pass subbands.
5. After getting the fused subbands, the inverse NSCT is applied and then the final fused image could be produced.

### 3.1. Novel Sum-Modified Laplacian (NSML)

Laplacian energy could well reflect the edge features of the low-pass subband images, so the NSML is employed in this paper as the external input of PCNN to improve the fusion performance. The definition of NSML is as

(2) Mi,j=2Ci,j−Ci−1,j−Ci+1,j+2Ci,j−Ci,j−1−Ci,j+1NSMLi,j=∑a ∑bWa,bMi+a,i+b2Wa,b=115121232121,

where *C*(*i*, *j*) means the coefficients of the low-pass subband image at (*i*, *j*). *a* and *b* denote the sizes of neighbor window which are generally 3 × 3, 5 × 5, and 7 × 7. In this paper, 3 × 3 is chosen for computing NSML. In addition, *W* means the weighted template, which emphasizes the coefficient at the center of window.

### 3.2. Energy of Edge (EOE)

Traditionally, the gray value of the original pixel is utilized as the input of PCNN which does not consider the effect of the neighborhoods. Moreover, it is known that human eyes are more sensitive to edge and directional and texture information rather than single pixel information. In order to make better use of the edge information, the EOE is selected as the external stimulation for PCNN model for high-pass subbands. The definition of EOE is shown as

(3) EOEi,j=∑i,j∈DWi,jLEi,jLEi,j=E1∗Ci,j2+E2∗Ci,j2+E3∗Ci,j2E1=−1−1−1222−1−1−1E2=−12−1−12−1−12−1E3=−10−1040−10−1,

where *W* is the weighted template. *D* denotes the neighborhood of (*i*, *j*). *C*(*i*, *j*) means the coefficients of the high-pass subband image at (*i*, *j*). *E*_1_, *E*_2_, and *E*_3_ mean the directional filtering operators. The EOE could well reflect the edge information in horizontal, vertical, and diagonal directions.

### 3.3. Region Energy

The rules of weighted average and larger absolute value are commonly used as the fusion rules to calculate the fused coefficients. However, these methods might lead to losing part of details of the source images and reducing the contrast. To improve the fusion performance, the relationships of the neighboring regions should be considered. In this paper, the rule of max region energy is adopted as the fusion rule. The definition of region energy is shown in (4) and (5):

(4) Ei,j=∑a ∑bwa,bC2i+a,j+b

(5) w1=1911111111

(6) DA,BFi,j=if EAi,j>EBi,jDBi,jif EAi,j≤EBi,j,

where *C*(*i*, *j*) means the coefficients of the firing map image at (*i*, *j*). *w*(*a*, *b*) is the energy template. Different energy templates are used for low- and high-pass subbands. *w*_1_ is the energy template used for low-pass subband images. And *w*_2_ is used for high-pass subbands to strengthen the coefficients at the window center. In addition, the rule of max region energy is defined as (6), where *D*_*A*,*B*_^*F*^ is the fused coefficient and *D*_*A*_ and *D*_*B*_ are the subband images which are obtained by employing NSCT on the source images *A* and *B*. *E*_*A*_ and *E*_*B*_ are the region energy for the corresponding firing maps of *A* and *B*, respectively.

### 3.4. Improved PCNN

The linking strength *β*, reflecting the variance of the coefficients of the subband images, plays a key role in fusion process. In traditional PCNN based fusion process, *β* is usually assigned manually according to experiences and all neurons in PCNN are set with the same linking strength value. However, according to [17], the values of *β* should not be all the same in different neurons. Besides, it has been verified that *β* is relevant to the image features of the corresponding pixels of the input images. If the external input coefficient is larger, then a higher value should be assigned to *β*. As a result, considering the edge information of the source image, the region average gradient is selected as the linking strength to improve its self-adaptability in this paper. The higher value of the average gradient, the higher clarity of the image. The definition of region average gradient is shown as

(7) βi,j=g−i,j=19·∑a=−11 ∑b=−11g1i+a,j+b+g2i+a,j+b21/2g1i,j=Ci,j−Ci+1,j2g2i,j=Ci,j−Ci,j+12,

where *C*(*i*, *j*) means the coefficients of the input image at (*i*, *j*). $\overset{-}{g}\left(i,j\right)$ is the average gradient at (*i*, *j*). The larger value of $\overset{-}{g}\left(i,j\right)$ means that the image has a higher clarity. Moreover, the higher the value of *β* is, the earlier the ignition of the correspondent neuron would be which will result in better use of the detailed information of the input image and increase the fusion effectiveness.

## 4. Results and Discussions

### 4.1. Experimental Data and Platform

To verify the effectiveness of the proposed fusion framework, we conduct experiments on whole-body PET/CT data which are provided by the General Hospital of Shenyang Military Area Command, Shenyang, China. All of our data is in conformity with laws and ethical standards. Each model of data consists of 239 slices. The original PET and CT images captured from devices are different from each other, such as the images sizes (the sizes of PET and CT images are 128 × 128 and 512 × 512, resp.) and the scale and geometric distortion, which would affect the effectiveness of fusion. Thus, it is necessary to do an interpolation and a registration process before fusion so that all PET images would be resized to 512 × 512 and the geometric displacement would be corrected. In this paper, we use the registration framework that we proposed in [22] to preprocess the PET and CT images before fusion. This framework could achieve a good performance on the registration of whole-body PET and CT images. The experimental platform is Intel® Core TM i7-2600 CPU @ 3.40 GHz, 8 G RAM, 1 T hard disk, Windows 7 OS. The integrated development environment is the MATLAB 2015b. Besides, the ITK-SNAP is utilized to view images in this paper.

### 4.2. Performance Measures

To quantitatively evaluate fusion performance of the proposed framework and the other compared methods, seven commonly used metrics are applied in this paper including average gradient (AG), Shannon entropy (EN), joint entropy (JE), cross entropy (CE), image quality index (IQI), *Q*_*E*_, and *Q*^*AB*/*F*^ [2, 10, 23].

The average gradient reflects the variance of the gray value which could be used to evaluate the clarity of one image. The higher value of gradient value denotes that the image is clearer and the fusion performance is better. The definition is shown as

(8) G−=1m−1n−1·∑i=1m ∑j=1nCi,j−Ci+1,j2+Ci,j−Ci,j+122,

where *C*(*i*, *j*) means the gray value of the input image at (*i*, *j*). *m* and *n* mean the size of the input image.

The Shannon entropy measures the information content in the source image. The higher value of Shannon entropy of the fused image, the more information it contained, the better fusion performance it has. The definition of Shannon entropy is shown as

(9) $$\begin{array}{c} EN=-\overset{L-1}{\underset{i=0}{\sum }}P_{i}\;\log_{2}\left(\;P_{i}\;\right), \end{array}$$

where *P*_*i*_ means the probability of the gray level *i* in the source image.

The joint entropy denotes the similarity of the fused image with the source images. The definition is as

(10) $$\begin{array}{c} H\left(\;F,A\;\right)=-\overset{L-1}{\underset{i,j=0}{\sum }}P_{i,j}\;\log_{2}\left(\;P_{i,j}\;\right), \end{array}$$

where *P*_*i*,*j*_ means the joint probability of the gray level *i* in image *F* and the gray level *j* in image *A*. The larger joint entropy denotes better performance.

The cross entropy measures the difference between the fused image and the source images. The cross entropy image fusion metric is defined as the average of the relative entropies between the source images *A* and *B* and the fused image *F*; see (11)

(11) CEA,B,F=DhA||hF+DhB||hF2Dp||q=∑i=0L−1pi log2⁡piqi,

where *D*(*p*||*q*) means relative entropy of two probability distribution functions *p*_*i*_ and *q*_*i*_. *H*_*A*_ and *H*_*B*_ are the normalized histograms of the source images *A* and *B*, respectively. The lower cross entropy means the better fusion performance.

The IQI between the fused image *F* and the source images *A* and *B* are defined as

(12) IQIA,B,F=IQIA,F+IQIB,F2

(13) IQIx,y=σxyσxσy·2x− y−x−2+y−2·2σxσyσx2+σy2,

where $\overset{-}{x}$,  *σ*_*x*_, and *σ*_*xy*_ are the mean of *x*, variance of *x*, and the covariance between *x* and *y*. If the IQI achieves higher values, it means the better fusion quality of the fused image.

*Q*_*E*_ is an edge-dependent fusion quality metric based on the IQI. The definition of *Q*_*E*_ is introduced as

(14) QEA,B,F=QwA,B,F·QwA′,B′,F′αQwA,B,F=∑w∈WcwλwIQIA,F ∣ w+1−λwIQIB,F ∣ wλw=sA ∣ wsA ∣ w+sB ∣ w,

where the definition of IQI is given in (13). *α* ∈ [0, 1] expresses the contribution of the edge images compared with the original images (usually *α* = 1). IQI(*A*, *F*∣*w*) and IQI(*B*, *F*∣*w*) denote the structural similarity measures between the source images and the fused image in a sliding local window *w*. *c*(*w*) is defined as *c*(*w*) = *C*(*w*)/∑_*w*∈*W*_*C*(*w*), where the overall saliency of a window *C*(*w*) is defined as *C*(*w*) = max{*s*(*A*∣*w*), *s*(*B*∣*w*)}. *s*(*A*∣*w*) and *s*(*B*∣*w*) denote saliency of image *A* and image *B* in window *w*. The larger *Q*_*E*_ means better fusion performance.

*Q* ^*AB*/*F*^ is a fusion performance metric based on the edge information to measure the similarity among images. The definition of *Q*^*AB*/*F*^ is defined as

(15) QAB/F=∑i=1M∑j=1NQi,jAFwi,jA+Qi,jBFwi,jB∑i=1M∑j=1Nwi,jA+wi,jBQi,jAF=Qg,i,jAF+Qα,i,jAFQi,jBF=Qg,i,jBF+Qα,i,jBF,

where *Q*_*g*,*i*,*j*_^·*F*^ and *Q*_*α*,*i*,*j*_^·*F*^ denote the edge strength and orientation preservation values, respectively. The larger value means the better fusion performance.

### 4.3. Results Evaluation and Discussions

To evaluate the performance of the proposed PET and CT fusion framework, we compare the fusion results of the proposed framework with the following six methods on the same dataset.

**Method 1 .**

It is DWT-based method where the fused coefficients are selected according to the rule of maximum absolute values.

**Method 2 .**

It is NSCT-based method (NSCT) where the maximum selection rule is used for both the low- and high-pass subbands [24].

**Method 3 .**

It is NSCT-PCNN based method (NSCT_PCNN_1) where the same rules with Method 2 are used here.

**Method 4 .**

It is image fusion method in the NSCT domain using spatial frequency-based PCNN method (NSCT-SF-PCNN) where the maximum selection rule is used for both the low- and high-pass subbands [25].

**Method 5 .**

It is an improved NSCT-PCNN based fusion method [10, 12, 14] named as NSCT_PCNN_2, where the NSML and modified spatial frequency (MSF) are as the external input of PCNN for low- and high-pass subbands, respectively.

**Method 6 .**

It is an improved NSCT-PCNN based fusion method [16] named as NSCT_PCNN_3, where EOE is as the external input of the PCNN.

**Figure 4:**
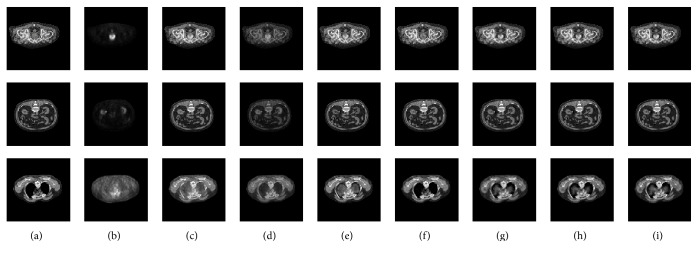
Three examples of the fusion results obtained by different methods. (a) CT images. (b) PET images. The (c)–(i) columns are fusion results produced by (c) DWT, (d) NSCT, (e) NSCT_PCNN_1, (f) NSCT_SF_PCNN, (g) NSCT_PCNN_2, (h) NSCT_PCNN_3, and (i) the proposed framework, respectively.

Figure 4 shows three examples of the fusion results obtained by these methods. (a) and (b) are the source CT images and PET images to be fused. (c)–(i) are the fusion results obtained by different compared methods. (c) shows the fusion results based on DWT. (d) presents the fusion results based on NSCT. (e) exhibits the fusion results produced by NSCT_PCNN_1. (f) shows the fusion results produced by NSCT_SF_PCNN. (g) and (h) are the fusion results produced by NSCT_PCNN_2 and NSCT_PCNN_3, respectively. (i) exhibits the fusion results produced by the proposed fusion framework. From the visual analysis of Figure 4, it is observed that the proposed framework could successfully preserve both the feature information of the CT images (e.g., the bony structures) and the PET images (e.g., high metabolic areas). Specifically, the fusion results produced by NSCT have the worst contrast and there are many artifacts introduced in the fusion results produced by DWT. Besides, the rest of the compared methods have similar performance with the proposed framework and it is difficult to determine which one is better by eyes.

**Figure 5:**
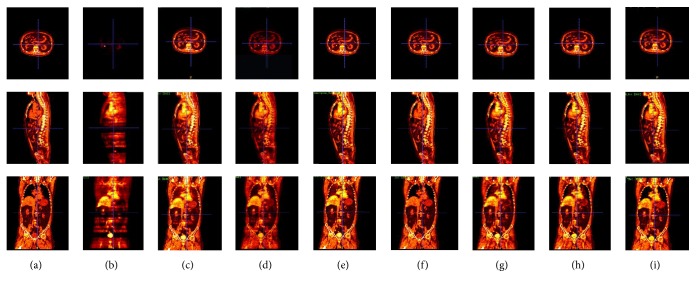
The 3D reconstructions with pseudo-color of the fusion result sequences. The first row represents transverse plane. The second row represents the sagittal plane. The last row represents the coronal plane. (a) and (b) are the 3D reconstructions of the source CT and PET image sequences, respectively. (c)–(i) are the reconstructions of the fusion result sequences that are produced by (c) DWT, (d) NSCT, (e) NSCT_PCNN_1, (f) NSCT_SF_PCNN, (g) NSCT_PCNN_2, (h) NSCT_PCNN_3, and (i) the proposed framework, respectively.

In order to evaluate the fusion results more intuitively, the 3D reconstruction is performed on the source data and the fusion result sequences (see Figure 5). In addition, the pseudo-color coding is also employed on these reconstructions for visualizing the results better. In Figure 5, the first row represents transverse plane of the reconstruction. The second row and third row represent the sagittal plane and the coronal plane, respectively. (a) and (b) are the reconstructions of the source CT and PET image sequences, respectively. (c)–(h) are the reconstructions of the fusion result sequences by DWT, NSCT, NSCT_PCNN_1, NSCT_SF_PCNN, NSCT_PCNN_2, and NSCT_PCNN_3, respectively. (i) is the reconstruction of the fusion results obtained by the proposed framework. From Figure 5, the fusion results obtained by the proposed framework could preserve both the structural feature information of CT and the functional feature information of PET. Besides, there are many artifacts in the results produced by DWT. Similar with Figure 4, the fusion results of NSCT are still the worst with the lowest contrast.

**Figure 6:**
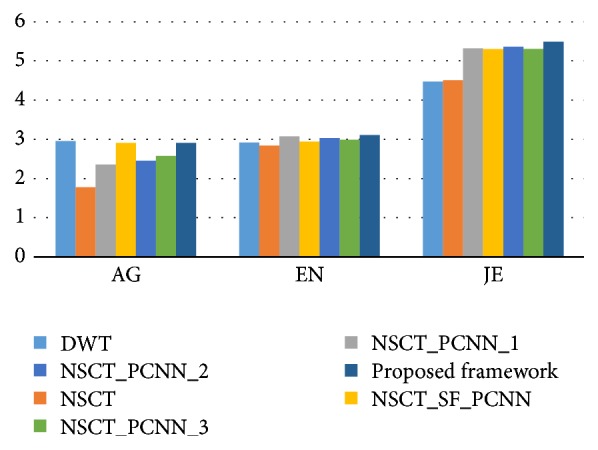
The average values of AG, EN, and JE of the compared methods.

**Figure 7:**
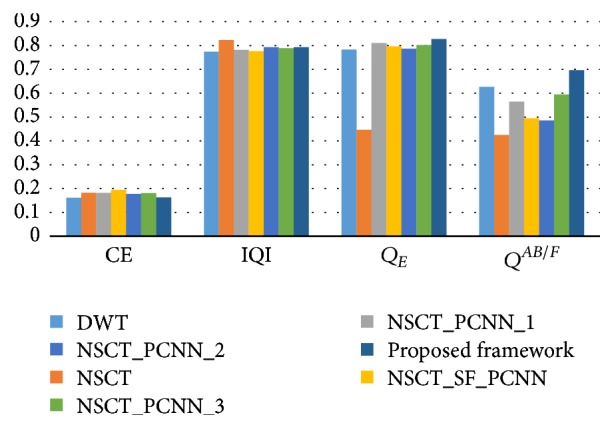
The average values of CE, IQI, *Q*_*E*_, and *Q*^*AB*/*F*^ of the compared methods.

According to Figures 4 and 5, it is difficult to distinguish which method achieves the best performance directly. In order to evaluate the fusion results between different algorithms objectively and quantitatively, this paper adopts seven metrics for performance evaluation which have been introduced in Section 4.2.  Table 1 shows the average values of these metrics on 239 pairs of PET and CT images. Figures 6 and 7 are the bar charts corresponding to Table 1. Note that the lower the value of CE, the better the fusion performance. The higher the values of the rest of metrics, the better the fusion performance. It can be seen that the performance of our proposed framework on these metrics is always on the top two among all algorithms which outperforms other methods. Although the DWT performs best on AG, CE, and *Q*^*AB*/*F*^, its values of EN, HE, and *Q*_*E*_ are worse than the others. In addition, the NSCT gives poorer results than other NSCT-PCNN based algorithms because PCNN could make use of the global feature information of the image which would improve the fusion performance. Following the analysis and discussion, a conclusion can be drawn that the proposed fusion framework outperforms the other image fusion methods for the fusion of whole-body PET and CT images. The fusion results of our framework could combine more information which is useful for diagnoses.

## 5. Conclusions

In this paper, a novel fusion framework based on adaptive PCNN in NSCT domain is proposed for fusing the whole-body PET and CT images. Our framework utilizes the average gratitude of each pixel as the linking strength in PCNN model to make it more adaptive. Besides, in order to make full use of the feature information contained by the input images, the NSML and EOE are as the linked input of the PCNN model in low-pass and high-pass subbands, respectively, to improve the fusion performance. Moreover, the rule of max region energy is adopted as the fusion rule and different energy templates are used for low- and high-pass subbands. The experiments on whole-body PET and CT images with well alignments demonstrate the good performance of the proposed framework. An evaluation on seven metrics including AG, EN, JE, CE, IQI, *Q*_*E*_, and *Q*^*AB*/*F*^ illustrates objectively that the proposed framework outperforms the other six methods.

## Figures and Tables

**Table 1:** The average fusion performances of different methods on PET/CT data.

Method	AG	EN	JE	CE	IQI	*Q*_*E*_	*Q* ^*AB*/*F*^
DWT	**2.95607**	2.91867	4.47628	**0.16158**	0.77437	0.78363	**0.62640**
NSCT	1.77727	2.84131	4.50450	0.18274	**0.82343**	0.44651	0.42512
NSCT_PCNN_1	2.35760	**3.07476**	5.32002	0.18201	0.78151	**0.81062**	0.56454
NSCT_SF_PCNN	2.90817	2.94637	5.30418	0.19419	0.77631	0.79662	0.49532
NSCT_PCNN_2	2.45416	3.03202	**5.36200**	0.17807	0.79298	0.78664	0.48583
NSCT_PCNN_3	2.58133	2.99083	5.30786	0.18161	0.78944	0.80226	0.59472
The proposed framework	**2.90951**	**3.11046**	**5.49125**	**0.16308**	**0.79330**	**0.82769**	**0.69649**
